# Engineering an Accurate Model of Portal Hypertension: Simulation With a Goal of Clinical Impact

**DOI:** 10.14309/ctg.0000000000000595

**Published:** 2023-09-25

**Authors:** Arun B. Jesudian

**Affiliations:** 1 Division of Gastroenterology and Hepatology, Weill Cornell Medicine, New York, New York, USA.

Clinically significant portal hypertension (CSPH) is widely accepted to be a prerequisite for decompensation in cirrhosis and further clinical deterioration. Although a hepatic venous pressure gradient (HVPG) ≥10 mm Hg constitutes CSPH, clinicians are well aware that 2 patients with similar HVPGs may manifest vastly different clinical consequences of their CSPH. For example, the presence of gastroesophageal varices is diagnostic of CSPH; however, up to 60% of patients with CSPH do not have varices (1). This discrepancy is postulated to be at least partially explained by the high prevalence of large and small spontaneous portosystemic shunts occurring in the setting of cirrhosis with portal hypertension (2).

In this issue of *Clinical and Translational Gastroenterology*, Mazmuder et al (3) harness computational modeling of hemodynamics, initially used for characterization the cardiovascular system, to enhance our understanding of the development and progression of portal hypertension in cirrhosis. They seek to optimize the clinical relevance of their model by incorporating variability in an individual's sensitivity to HVPG changes as determined by the ease with which their venous system remodels leading to shunt formation—a phenomenon that may well explain the disparities in clinical presentations of patients with similar degrees of portal hypertension. They further investigate both the trajectory of HVPG over time and the predicted response to transjugular intrahepatic portosystemic shunt insertion while accounting for this heterogeneity in sensitivity to venous remodeling.

By running numerous simulations on their model (which the reader is invited to interact with firsthand https://filip-jezek.github.io/Ascites/), the authors reached several conclusions. First, the increase in portosystemic gradient (approximated by HVPG) varied based on patients' sensitivity in terms of capacity to remodel and shunt. Interestingly, gradients rose in a nonlinear manner and, perhaps more predictably, were lower in those who shunted more. When compared with patients insensitive to venous remodeling, those with more propensity to shunt were noted to have higher shunt flow, lower HVPG, and earlier varices combined with later ascites formation. Finally, the model observed that patients with more shunting pre-transjugular intrahepatic portosystemic shunt experienced a less pronounced reduction in portosystemic gradient and a lower proportional decrease in residual portal venous flow.

There are certainly limitations on the extent to which clinical conclusions can be drawn from this computer simulation of portal system hemodynamics. The inputs into the model were not obtained directly from patient-level data, a consideration which must be kept in mind when interpreting the results and assessing their reliability in clinical practice. In addition, this early version of the model studies the portal system in isolation. However, the true hemodynamics of *in vivo* portal hypertension are considerably more complex. These can be influenced by changes in systemic circulation, most notably cirrhotic cardiomyopathy and renal dysfunction, which are not accounted for in the current simulation (4). In addition, the present model is further limited in that the splanchnic inflow is constant, whereas portal flow is known to increase as a result of splanchnic arteriolar vasodilation in the setting advanced portal hypertension (5). The authors comment that their future work seeks to incorporate whole-body models into a more physiologic simulation of portal hypertension.

As our understanding of the clinical ramifications of CSPH progresses, it is important to focus on individualizing our therapeutic approach based on unique patients' needs. Mazmuder et al, through their engineered computational model of portal hypertension, bring us 1 step further to realizing that goal of personalized management of complications of portal hypertension.

## References

[1] Garcia-Tsao G Abraldes JG Berzigotti A . Portal hypertensive bleeding in cirrhosis: Risk stratification, diagnosis, and management: 2016 practice guidance by the American Association for the study of liver diseases. Hepatology 2017;65(1):310–35. 27786365 10.1002/hep.28906

[2] Simón-Talero M Roccarina D Martínez J . Association between portosystemic shunts and increased complications and mortality in patients with cirrhosis. Gastroenterology 2018;154(6):1694–705.e4. 29360462 10.1053/j.gastro.2018.01.028

[3] Mazmuder N Jezek F Tapper EB . Portal venous remodeling determines the pattern of cirrhosis decompensation: A systems analysis. Clin Transl Gastroenterol 2023. [Epub ahead of print April 22, 2023.] 10.14309/ctg.0000000000000590 PMC10522110 37092902

[4] Krag A Gluud LL. Cross-talk between the liver, heart and kidney: Another piece in the puzzle. J Gastrointestin Liver Dis 2014;23(2):119–21. 24949601 10.15403/jgld.2014.1121.ak1

[5] Engelmann C Clària J Szabo G . Pathophysiology of decompensated cirrhosis: Portal hypertension, circulatory dysfunction, inflammation, metabolism and mitochondrial dysfunction. J Hepatol 2021;75(Suppl 1):S49–66. 34039492 10.1016/j.jhep.2021.01.002 PMC9272511

